# Modelling of macrophage responses to biomaterials *in vitro*: state-of-the-art and the need for the improvement

**DOI:** 10.3389/fimmu.2024.1349461

**Published:** 2024-03-26

**Authors:** Svetlana Piatnitskaia, Guzel Rafikova, Azat Bilyalov, Svyatoslav Chugunov, Iskander Akhatov, Valentin Pavlov, Julia Kzhyshkowska

**Affiliations:** ^1^ Cell Technology Laboratory, Institute of Fundamental Medicine, Bashkir State Medical University, Ufa, Russia; ^2^ Additive Technology Laboratory, Institute of Fundamental Medicine, Bashkir State Medical University, Ufa, Russia; ^3^ Laboratory of Immunology, Institute of Urology and Clinical Oncology, Bashkir State Medical University, Ufa, Russia; ^4^ Laboratory of Mathematical modeling, Institute of Fundamental Medicine, Bashkir State Medical University, Ufa, Russia; ^5^ Institute of Urology and Clinical Oncology, Department of Urology, Bashkir State Medical University, Ufa, Russia; ^6^ Laboratory for Translational Cellular and Molecular Biomedicine, Tomsk State University, Tomsk, Russia; ^7^ Institute of Transfusion Medicine and Immunology, Mannheim Institute of Innate Immunosciences (MI3), Medical Faculty Mannheim, Heidelberg University, Mannheim, Germany; ^8^ German Red Cross Blood Service Baden-Württemberg—Hessen, Mannheim, Germany

**Keywords:** implants, inflammation, cytokine, healing, fibrosis, monocyte

## Abstract

The increasing use of medical implants in various areas of medicine, particularly in orthopedic surgery, oncology, cardiology and dentistry, displayed the limitations in long-term integration of available biomaterials. The effective functioning and successful integration of implants requires not only technical excellence of materials but also consideration of the dynamics of biomaterial interaction with the immune system throughout the entire duration of implant use. The acute as well as long-term decisions about the efficiency of implant integration are done by local resident tissue macrophages and monocyte-derived macrophages that start to be recruited during tissue damage, when implant is installed, and are continuously recruited during the healing phase. Our review summarized the knowledge about the currently used macrophages-based *in vitro* cells system that include murine and human cells lines and primary ex vivo differentiated macrophages. We provided the information about most frequently examined biomarkers for acute inflammation, chronic inflammation, foreign body response and fibrosis, indicating the benefits and limitations of the model systems. Particular attention is given to the scavenging function of macrophages that controls dynamic composition of peri-implant microenvironment and ensures timely clearance of microorganisms, cytokines, metabolites, extracellular matrix components, dying cells as well as implant debris. We outline the perspective for the application of 3D systems for modelling implant interaction with the immune system in human tissue-specific microenvironment avoiding animal experimentation.

## 1 Introduction

Implants is essential line for therapy in reconstructive and regenerative medicine. Biomaterials for implants construction are constantly under development with the aim to adjust them to the tissue-specific and disease specific conditions, to enhance their biocompatibility, support healing and healthy long-term integration. The physical, chemical and bioactive characteristics of biomaterials have significant impact on the spectrum and scale of tissue responses during acute inflammatory phase that accompany initial installation of the implant. Jamieson et al. found that Al_2_O_3_ or ZrO_2_ ceramic particles can induce IL-1β, IL-8, CCL2, CCL3, CCL4 (C-C motif) ligand)) production in monocyte-like THP1 cells, and toll-like receptor 4 (TLR4) was found to be a principal receptor for this effect (1). The effect of different metal particles of Co-Cr-Mo alloy on macrophages has been shown to induce increased macrophage activity and production of M1-type inflammatory cytokine IL-1β (2). Lei Sun’s et al. investigated role of magnesium in modulating the behavior of macrophages (3). They exposed THP-1 cells to the various concentrations of magnesium and examined the changes in their phenotype. Expression of pro-inflammatory cytokines (tumor necrosis factor alfa (TNF-α) and IL-1 β) was significantly downregulated by magnesium in a time-dependent manner. Magnesium ions were also able to shift THP-1 cells towards M2 phenotype, characterized by enhanced secretion of anti-inflammatory cytokine IL-10 (3).

This influence continues into the resolution of inflammation/healing phase. Moreover, these characteristics play a crucial role in the long-term period, ensuring that an ideal implant does not induce specific tissue reactions and is fully integrated into the local tissue microenvironment. Physical and chemical characteristics of the implant are measurable, and precise methodology is well-established. At the level of the interaction with the biologicals systems, most frequently toxicity of dividing cells and effects of implants on the osteo-integration are examined, while the reaction of immune system on the implant materials are frequently underscored or even neglected. Each tissue in our body is equipped with the natural defense mechanism against foreign substances, where key cells in the foreign body reaction are resident tissue macrophages, that are actively control healthy tissue homeostasis and turnover (4–6).

The properties of biomaterials can promote spectrum immune responses, causing increased inflammation, impaired healing, promotion of fibrotic encapsulation, and tissue destruction, which becomes a cause of implant complications such as peri-implant inflammatory response and implant instability. Various cells such as monocytes/macrophages, dendritic cells (DCs), and neutrophils are involved in biomaterial-induced tissue remodeling that can results in scar formation or loss of function, as well as in the development of chronic inflammatory response, non-healing wounds, fibrosis, and implant failure (7, 8).

Macrophages and neutrophils perform both phagocytic and signaling functions, especially in the initial inflammatory phase of biomaterial implantation. These cell types ultimately determine the outcome of implants in the form of chronic inflammatory response, fibrosis or integration. Other cell types such as DCs, mastocytes, natural killer cells, and intrinsic lymphoid cells may also play an immunomodulatory role in the context of biomaterial implantation (9).

Macrophages are essential innate immune cells that are present in all adult tissues, and dynamically control tissue homeostasis and healthy tissue turn-over (10). In response to trauma and pathogen attack, resident tissue macrophages provide first line defense against the danger, and signal to other innate and adaptive immune cells to be recruited to the site of tissue damage from blood circulation. During the acute inflammatory phase macrophages will primarily secrete the anti-bacterial agents (reactive oxygen species (ROS), lysosomal enzymes) and inflammatory cytokines to amplify the reaction of other immune cells (11, 12). Macrophages have the ability to sense the microenvironmental signals once pathogen attack is eliminated, and to switch on the program of the resolution of inflammation followed by the health phase. At this stage the major activity of macrophages will include release of anti-inflammatory cytokines, extracellular matrix components, induction of somatic cell proliferation and vascularization, and clearance of the debris using scavenging receptors (13). Macrophages have an intrinsic ability to complete the healing phase and to restore the dynamic tissue homeostasis. This ability of macrophages to orchestrate the defense and healing processes in multiple tissues is based on their plasticity in response to changing content of the stimuli (14, 15). However, such fine-tuning can be disturbed by metabolic alterations and by foreign bodies, including implanted biomaterials (5, 16–18). Efficiency of macrophages action depends not only on the resident tissue macrophages, but also on the programming of their pre-coursers circulating monocytes, that are continuously produced by bone marrow and are massively recruited to the damaged tissue already within first hour of the traumatic injury or surgery (19–21). Inflammatory programming of monocytes can be caused by pre-existing infections, exposure to pollution, metabolic disorders or therapeutic interventions. Such inflammatory programs can be detected on transcriptional and epigenetic levels, and will interfere with efficient implant integration.

In this review, focus on the essential steps and processes in implant interaction with resident tissue macrophages, present the state-of-the art in the *in vitro* or ex vivo modelling of implant/macrophage interactions, and highlight the perspective of developing 3D models to assess macrophage reaction on newly developed biomaterials with high immunocompatibility score.

## 2 Implant materials and inflammation

### 2.1 Acute inflammation and the foreign body response

Implantation is always associated with surgical injury and biomaterial implantation will induce a classic pathophysiological acute inflammatory response. In addition, due to their size, shape, surface morphology and chemical properties, biomaterial implants are recognized by the immune system (including bot resident and newly attracted immune cells) as foreign bodies and induce a foreign body reaction (FBR), with the clinical manifestations of reactions depending on the type of implant, its location and individual health status of a patient (22).

In the early stages of implantation, the interaction between blood and biomaterial initiates with protein adsorption on the biomaterial surface and the formation of a temporary matrix, incorporating fibrin (22). This transient matrix provides structural and biochemical components for wound healing and responses to foreign bodies. The chemoattractive properties of chemokines, such as transforming growth factor-beta (TGF-β), platelet-derived growth factor (PDGF), CXCL4, leukotriene B4 (LTB4) and IL-1, attract macrophages to the implantation site (23). Degranulation of mast cells and histamine release also contribute to this process. Macrophage assembly around the implant leads to further recruitment of macrophages, which produce various cytokines, including PDGF, TNF-α, IL-6, G-CSF, and GM-CSF, intensifying inflammatory reactions and foreign body responses. Chemokines like CCL2, CCL4, CCL13, and CCL22 can additionally attract supplementary macrophages to the implantation site. Macrophages arriving at the implantation site may adhere and participate in subsequent FBR and wound healing, transitioning to subsequent phases of inflammation (24). Implants can also induce an inflammatory response through the activation of receptors expressed in both immune and non-immune cells (25). These receptors recognize endogenous signals activated during cell injury. These receptors include Toll-like receptor (TLR), C-type lectin receptor (CLR), retinoic acid inducible gene (RIG)-I-like receptor (RLR) and NOD-like receptor (NLR), IL-1 receptor (IL-1R), IL-6 receptor (IL-6R) and TNF receptor (TNFR). Signaling through these receptors activates an intracellular signaling cascade that leads to nuclear translocation of transcription factors such as activator protein-1 (AP-1) and NF-κB or interferon regulatory factor 3 (IRF3). Stimuli activate immune cells such as macrophages and induce the production of inflammatory cytokines such as IL-1β, IL-6, TNF-α as well as inflammatory proteins and enzymes (25).

The immediate post-implantation response is characterized by precipitation of circulating proteins such as albumin, fibrinogen, fibronectin, gamma globulins, platelet aggregation, activation of the complement system and development of a provisional matrix (26–28). The predominant component of the provisional matrix is fibrin. It has been found that traumatic injury induced by biomaterials installation drives polymerization of fibrin, a major component of blood cloths, on the implant surfaces. This process can lead to the inflammatory response, as the immune system recognizes the opsonized surface the implant as dangerous object (29). In a study of early reactions to a foreign body (FB) in mice, it was found that fibrinogen deficient mice prevented a normal inflammatory reaction until the implant was coated with this protein (26).

**Figure 1:**
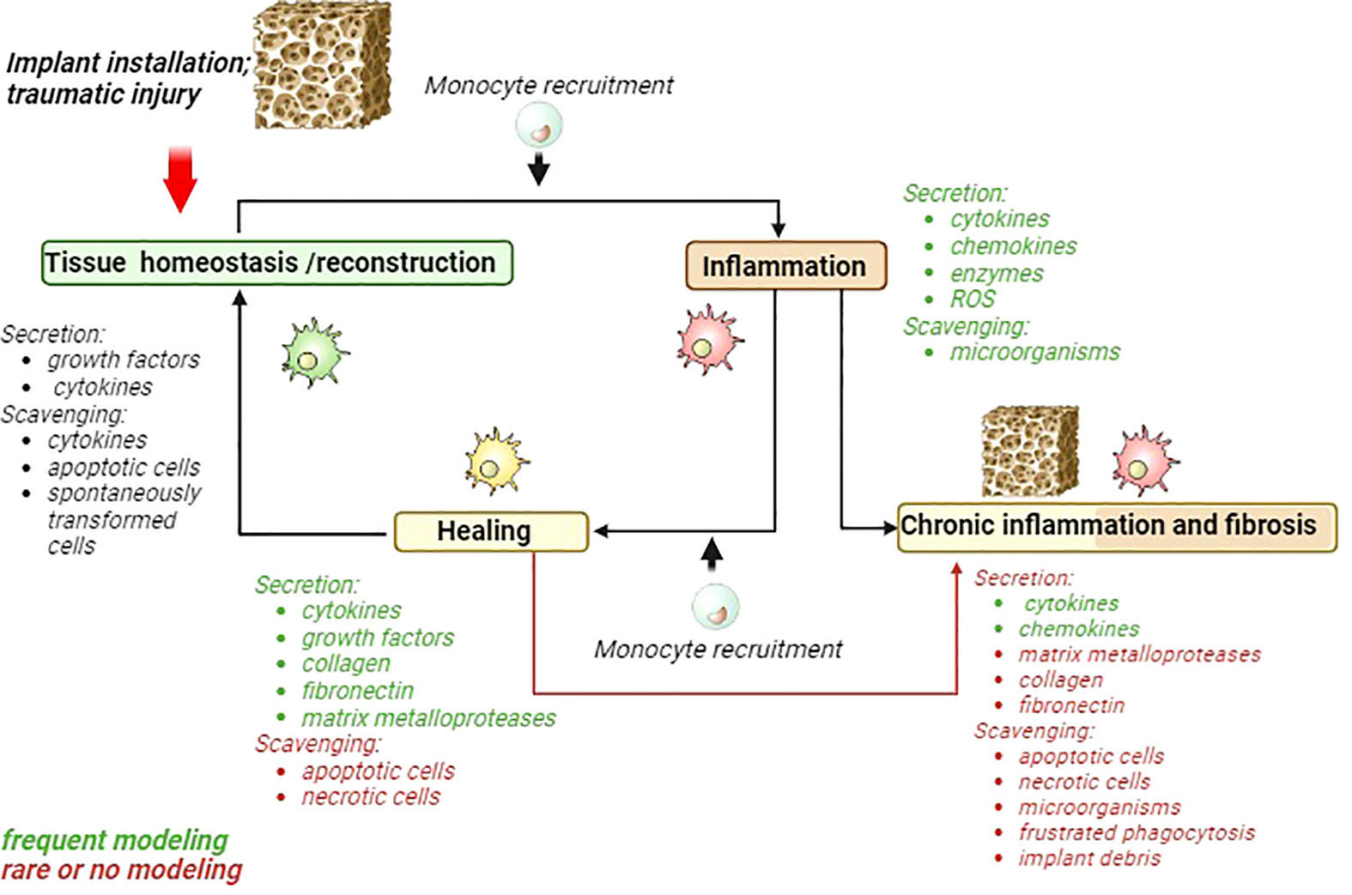
Schematic presentation of acute inflammatory cycle and deviations towards chronic inflammation and fibrosis. Major macrophages activities are listed for each phase. Activities frequently modeled in 2D models are marked green. Activities that are rarely modeled or almost ignored are marked in red. MMP, matrix metalloproteinases; ROS, reactive oxygen species.

Types of immune cells that are involved in the recognition and responses to implants are summarized in **Table 1**. By investigation of the biomaterials interaction with immune cells, most commonly pro-inflammatory and anti-inflammatory cytokines are taken under consideration, while several essential functions of innate immunity that include clearance of bacteria, tissue debris and apoptotic cells, and necrotic cells do not attract the necessary attention (**Figure 1**).

**Table 1:** Innate immune cell mediators in response to implants.

Cells	Acute phase mediators	Healing phase mediators	Long-term complicaitons mediators	Frustrated phagocytosis	References
Innate immune cells					
**Monocytes/macrophages**	TNF-α IL-1β IL-6 IL-8 CXCL9 CXCL10 CXCL11	IL-1Ra IL-4 IL-10 IGF-1 VEGF PDGF TGF-β	PDGF TGF-β	TNF-α IL-1β IL-6 IL-4 IL-10	(30–39)
**Neutrophils**	ROS NO IFN-γ	MMPs	Not found	ROS	(40–42)
**Basophils**	Not found	Not found	Not found	Not found	
**Eosinophils**	Not found	Not found	Not found	Not found	
**Mast cells**	Histamine	Not found	Not found	Not found	(43, 44)
**NK**	TNF-α IL-2 CCL3 (MIP-1α)	CXCL7,suggested role	Not found	Not found	(45, 46)

In our review we focused on macrophages, key cells that orchestrate inflammation, healing and FBR. Implantation induces recruitment of circulating monocytes and responses of resident tissue macrophages in all tissue types. Upon recognition of a foreign substance, macrophages migrate and adhere to the implant surface (47). The interaction of macrophages with the substrate is mediated by cellular receptors for integrin proteins such as CR3, avß3, a5ß1. Acute inflammatory stage, where macrophages produce TNF-α, IL-1β, IL-6 is followed by resolution and clearance stage and matrix reconstruction stages, that can also lead to tissue fibrotization (5, 48, 49).

In attempt to engulf a foreign body, macrophages can attach to the surface of implant and fuse to form multinucleated cells foreign body giant cells (FBGCs). FBGCs function as macrophages display the ability to phagocytose, to generate oxygen radicals and nitrogen, to produce cytokines and growth factors. High concentration of growth factors, such as TGF-β, around the biomaterial contributes to the transformation of fibroblasts to myofibroblasts (50). In addition, macrophages can promote osteogenesis in the early and middle stages without enhancing matrix mineralization by induction of the expression of BMP-2, RUNX2 (51, 52).

Taken together, macrophages contribute to the necessary inflammatory response, but prolonged pro-inflammatory activation of macrophages leads to the detrimental in case of implant FBR granuloma formation and fibrosis, resulting in chronic inflammation and failure of biomaterial integration.

### 2.2 Healing phase and frustrated phagocytosis

Macrophages at healing stage are essential for the resolution of inflammation, abrogation of unnecessary immune infiltrate accumulations, scavenging of cell and matrix debris, support of somatic cell proliferation and extracellular matrix reconstruction (13). Macrophage plastic transition from M1 to anti-inflammatory M2 state is pre-requisite for the start of tissue healing, which is needed for the integration of implants (53). M1 and M2 definitions, previously used to describe subpopulations of macrophages, are now mostly applied to indicate the vectors of macrophage polarization toward acute inflammatory rarefactions (M1) or towards healing; anti-inflammatory, homeostatic or tolerogenic phenotypes, that are needed for effective tissue regeneration, but are detrimental in case of cancer (54). M2 macrophages express elevated levels of diverse, tissue specific scavenger receptors, including CD68, CD163, CD206, MARCO, CD36 (55). The common functional feature of SR is internalization molecules, molecular complexes and larger particles and targeting them for lysosomal degradation (56). Major pathway for the SR-mediated internalization include endocytosis and phagocytosis, that in healthy adult tissue are essential for the dynamic tissue homeostasis and clearance of apoptotic bodies, components of extracellular matrix and metabolic ligands. The need for the scavenging function is significantly increased during the phase of resolution of inflammation and healing when amount of apoptotic bodies and unwanted molecules is increased, and macrophage scavenging function will provide the necessary clearance and reconstitution of healthy tissue composition. However, the tolerogenic scavenging activity of macrophage can be converted to the detrimental low grade inflammatory activity by the interaction with implant materials, since scavenger receptor can cooperate with other types of receptors (for example TLRs) and can drive inflammatory signal transduction and cytokine release (57–59). Thus outcome of different SR activities will be the decision of monocytes and macrophage to guard homeostatic balance, or to create chronic inflammatory microenvironment that frequently is further complicated by fibrosis (56). Scavenger receptor on macrophages play an essential role in clearing and removing not only of cellular debris, apoptotic cells, but also fibrin, and growth factors, including epithelial growth factor (EGF) and GDF-15 (55, 60–62). To carry out this analysis, an *in vitro* endocytosis or phagocytosis tests can be used. *In vivo* and *in vitro* tests for phagocytosis are essential for understanding the functionality of the immune system, evaluating the effectiveness of potential therapeutic agents, and studying diseases associated with immune dysfunction (63). The choice between *in vivo* and *in vitro* testing depends on specific research objectives and the complexity of the immune response under investigation.

*In vivo* phagocytosis tests are conducted within a living organism, typically in an animal model (64). The selection of the model depends on specific research requirements. *In vivo* phagocytosis tests are valuable for comprehending the overall immune response in an organism, simulating a more intricate physiological environment (64, 65). Thus, the study of phagocytosis *in vivo* was carried out by injecting S. cerevisiae labelled with Congo red into the coelomic cavity of A. altiparanae, then the phagocytic index was measured in fish blood. In addition, phagocytosed and non-phagocytosed yeasts were detected by optical microscopy analysis due to Congo red labelling (66).

*In vitro* phagocytosis tests can be performed in controlled laboratory conditions outside a living organism (67). *In vitro* phagocytosis tests are useful for dissecting specific cellular mechanisms involved in phagocytosis and provide a controlled environment for studying interactions between phagocytes and foreign particles (68, 69). Thus *in vitro* tests allowed us to analyze the phagocytosis of human monocytes cultured with plain latex beads or FITC-labelled Escherichia coli in classically or alternatively activated macrophages (70). The research of phagocytosis function using donor-derived human monocytes allowed us to evaluate the interaction between phosphatidylserine and stabilin-1, and to determine the function of stabilin-1 on alternatively activated macrophages (71). An *in vitro* study by Onyishi et al. investigated the role of TLR4 in phagocytosis, where they found that loss of TLR4 function increased phagocytosis of unopsonised cryptococci by murine and human macrophages (72).

These tests play a crucial role in advancing our understanding of immune responses and are instrumental in the development and assessment of therapeutic interventions for immune-related disorders. The selection of the testing approach depends on the specific nuances of the research goals and the desired level of experimental control.

However, scavenging function is almost neglected by testing effects of biomaterials with immune system (**Figure 1**).

Biomaterials can influence the adhesion and migration of monocytes and macrophages, which affects inflammation and regeneration processes. Tests on cell cultures have shown that the composition of the extracellular matrix and adhesion geometry can influence the shape and function of macrophages (73). *In vitro* tests of mesoporous silica with D(L)-mannose modified surfaces showed that the number of macrophages that attached to the modified surface was about four times higher than to the unmodified surface (74). Tests on the macrophage cell line J774A.1 for pure titanium with polished or grained surfaces showed increased adhesion for rough surfaces (75). Therefore, selection of biomaterials for the implants or developing of new biomaterials requires consideration of their effects on the adhesion of monocytes and resident tissue macrophages.

Majority of studies that model macrophages interaction with biomaterials assess cytokine production of gene expression and secreted levels. During the healing phase macrophage secreted number of cytokines, including IL-1Ra, IL-10, VEGF, PDGF, and TGF-β, which facilitate cell proliferation, osteochondral differentiation, and angiogenesis (76). Frequently applied methods include ELISA, PCR and flow cytometry to quantify production of cytokine and growth factors. Other mediator needed for healing phase include collagen, fibronectin and matrix metalloproteases (MMPs) (**Figure 1**).

Proinflammatory activation of macrophages directly contacting with the implant surface results in frustrated phagocytosis, leading to local inflammation. This process is often observed in the context of medical implants, where macrophages attempt to engulf the implanted material, but fail due to the size of the implant (5). The presence of frustrated macrophages producing ROS and degradative enzymes may lead to chronic inflammation, fibrosis, and implant instability, which are undesirable for tissue repair and integration (40).

State-of-the art approaches, such as spatial transcriptomics, enable not only identifying immune cell types that migrate to the site of injury but also determinate their spatial distribution in relation to the injury site and to each other. Foster et al.’s study on wound healing in the Rainbow mouse model, with implanted stents, analyzed postoperative days (POD) 2, 7, and 14, with the undamaged skin serving as the control. Using the 10× Genomics Visium method, the study demonstrated the highest levels of activated macrophage markers, such as Msr1, in the wound’s central region at POD 7 (77). The study conducted by Theocharidis et al. investigated wound and peri-wound tissue in patients with both healing and non-healing diabetic foot ulcers. The authors employed spatial single-cell transcriptomic analysis using NanoString’s GeoMx Digital Spatial Profiling platform to reveal that patients with healing wounds exhibited an increased polarization of M1 macrophages, naive and central memory T-cells, while patients with non-healing ulcerative defects had higher levels of M2 macrophages and NK-cells (78). Evident expression of Notch2 around the implanted biomaterial was detected. Selective inhibitors of Notch signaling pathways effectively decreased M1-like macrophages and stimulated M2-like macrophages, through the support of the scaffold (79). Gong et al. investigated the spatial transcriptomics of glial scar formation in spinal cord injury in mice and identified four possible phases of scar formation: macrophage infiltration, proliferation and differentiation of scar-resident cells, scar appearance and the stable scar (80). The primary cell types identified in the scar were microglia, macrophages, astrocytes, oligodendrocytes, fibroblasts, and endothelial cells. Heterogeneity was observed within the macrophage population, and subsequently three subpopulations were identified. The first subpopulation, making up 45.3% of all macrophages, showed high expression of lysozyme (Lyz2), a specific marker for macrophages, as well as thyrotropin-releasing hormone. The second subpopulation, comprising 51.8% of macrophages, exhibited elevated levels of platelet factor 4 (PF4). The third subpopulation comprised 2.9% of the macrophages and exhibited moderate levels of Lyz2 and low levels of PF4. Interestingly, macrophages belonging to subpopulation 2 consistently resided in the central region of the injury site, while those from subpopulation 1 surrounded them. Macrophages from subpopulation 3 were exclusively observed at the periphery of the injury site. The quantity of cells in subpopulation 2 remarkably lowered, while the quantity of cells in subpopulation 3 significantly increased. Of particular importance was the observation that cells from subpopulation 3 mixed with cells from subpopulation 1, creating a circular band of cells. The expression of the macrophage marker PF4 was significantly increased after 3 and 7 days and returned to baseline levels at the intermediate stage (80). Thus, macrophages have a dynamic changes of their activity during the healing process, and such dynamics has to be taken under consideration by modeling of biomaterials/macrophage interactions.

### 2.3 Chronic inflammation and fibrosis

Many type of cells are involved in various inflammation phases, however macrophages play key regulatory roles at all stages of inflammation initiation and resolution. In chronic inflammation, the interaction between collagen, fibrin, and macrophages plays a crucial role in the pathological process. Collagen and fibrin are key components of the extracellular matrix (ECM)and thrombotic clotting, respectively. M1 macrophages are able to start and sustain inflammatory responses, secreting pro-inflammatory cytokines, activating endothelial cells, and inducing the recruitment of other immune cells into the inflamed tissue; on the other hand, M2 macrophages promote the resolution of inflammation, phagocytose apoptotic cells, drive collagen deposition, coordinate tissue integrity, and release anti-inflammatory mediators (81).

The study on the impact of ECM modification using carboxyethylpyrrole (CEP) on the adhesive properties of M1-polarized macrophages, especially during chronic inflammation, has revealed mechanisms in the context of inflammatory reactions (82). *In vitro* experiments using BSA revealed that CEP can modify 10-20 lysines within a single protein molecule. Importantly, this modification involves the substitution of positively charged lysines with pyrroles, exposing negatively charged carboxyl groups. Recent findings from the research indicate that the carboxyl group within the CEP structure plays a specific role in binding to integrins αDb2 and αMb2 (82). It was found that CEP modification of ECM proteins, such as collagen IV and laminin, enhances their adhesive properties to M1 macrophages, particularly through integrin αDβ2. This contributes to the retention of M1 macrophages at the site of inflammation and may be associated with detrimental processes during chronic inflammation, autoimmunity, and other pathological conditions (82).

The impact of tissue density changes on macrophage activation and functions remains poorly understood. In the study conducted by Sapudom et al., THP-1 monocytic cells were incorporated into three-dimensional collagen matrices with varying fibril density and differentiated into macrophages using PMA (83). Subsequent activation (MLPS/IFNγ and MIL-4/IL-13) induced differences in cytokine secretion profiles, favoring IL-1β and TNFα in MLPS/IFNγ and IL-6 in MIL-4/IL-13. Notably, cytokine secretion increased with higher fibril density (40). It was found that M1LPS/IFNγ enhanced monocyte tissue infiltration, while MIL-4/IL-13 supported fibroblast differentiation into myofibroblasts via TGF-β1, depending on fibril density, indicative of an M2a-like phenotype (83).

In study Hsieh et al. have observed that fibrin and its precursor, fibrinogen, elicit distinctive functions in macrophages (84). When macrophages were cultured on fibrin gels created by combining fibrinogen with thrombin, it stimulated the secretion of the anti-inflammatory cytokine, IL-10. In contrast, exposing macrophages to soluble fibrinogen led to a significant increase in the production of the inflammatory cytokine, TNF-α. Importantly, when macrophages were cultured on fibrin gels in the presence of soluble fibrinogen, they maintained their anti-inflammatory characteristics. Additionally, adhesion to fibrin matrices inhibited TNF-α production in response to stimuli such as LPS and IFNγ, well-known for promoting inflammatory macrophage polarization (84). These findings reveal that fibrin plays a protective role in macrophage function, preventing their inflammatory activation triggered by various factors, including fibrinogen, LPS, and IFN-γ. This study suggests that the presentation of fibrinogen could serve as a critical regulator of macrophage behavior, offering a valuable immunomodulatory strategy for tissue healing and regeneration.

In the study Rudnic et al. focused on systemic sclerosis (SSc), an autoimmune disease characterized by excessive ECM production and multiorgan fibrosis (85). The role of monocytes and macrophages in SSc fibrogenesis was unclear. Immunohistochemistry found CD14+ monocytes in collagen-rich areas, along with alpha-SMA+ fibroblasts, CD68+, and man-nose receptor+ macrophages in SSc patients’ hearts and lungs. CD14+ monocyte transcriptomics revealed dysregulation in cytoskeleton, ECM, FN1 gene, and TGF-β signaling. Single-cell RNA sequencing showed activated profibrotic signature in CD14+ pulmonary macrophages from SSc patients with lung disease. Profibrotic cytokine exposure increased type I collagen, fibronectin, αSMA in CD14+ monocytes. Co-culture with dermal fibroblasts amplified profibrotic markers. TGF-β pathway inhibitors reduced fibronectin and collagen secretion. The study provided evidence for CD14+ monocytes/macrophages as ECM producers (85).

The cytokine and chemokines parameters are often examined in the of chronic inflammation phase (**Figure 1**). The collagen, fibronectin, MMPs and scavenging function apoptotic, necrotic cells, microorganisms, frustrated phagocytosis and implant debris are underscored in their significance (**Figure 1**).

Acute and chronic inflammation can lead to fibrosis, with macrophages playing a crucial role in fibrotic processes through the release of mediators such as TGF-β1 and PDGF. These mediators contribute to fibroblast migration, proliferation, and collagen synthesis, thereby promoting fibrosis (86, 87). Macrophages also play a vital role in the production and regulation of matrix MMPs, enzymes responsible for degrading various components of the ECM. Matrix metalloproteinases are produced by immune cells, fibroblasts, endothelial cells, osteoclasts (88). In our manuscript, we focused on macrophages MMPs, which play a role in ECM remodeling by degrading ECM and promoting angiogenesis and tissue remodeling.

The production of MMPs is activated in response to pathogens, TNF-α and other inflammatory mediators, with different MMPs playing different roles in the inflammatory response (89). In particular, MMP-9 has been implicated in tissue damage and biomaterial degradation (90). Reiss et al. established a murine wound model to investigate the effect of MMP-9 on chronic wound healing and demonstrated a delayed healing process in the presence of MMP-9 (91). MMP-12, also known as macrophage elastase, is a zinc-dependent protein that is critical for tissue remodeling (13). Madala et al. found that MMP-12 deficiency leads to increased expression of other ECM-degrading MMPs such as MMP-2, MMP-9 and MMP-13. This upregulation of MMP expression may limit the degradation of ECM components, thereby reducing the development of fibrosis (92). Stawski et al. utilized MMP12KO mice to assess the contribution of MMP-12 to vascular injury and fibrosis in the angiotensin II (Ang II) model (93). They observed that MMP-12 deficiency inhibited Ang II-induced production of TGF-β1, a profibrotic mediator, in the skin and perivascular regions of the heart. In Ang II-infused MMP12KO mice, TGF-β1-positive cells were significantly decreased in the perivascular regions of the heart but not in the interstitium (93).

Chitinases, including Chitinase 1 (CHIT1), chitinase 3-like-1 (CHI3L1) are implicated in the regulation of fibrosis, a pathological condition characterized by the excessive accumulation of ECM proteins leading to scarring and organ damage (94, 95). Lee et al. demonstrated that CHIT1 is required for the development of pulmonary fibrosis, and TGF-β1 plays a critical role in this process (94). They used wild type and CHIT1 null mutant mice, and demonstrated that TGF-β1 stimulated the production of ECM proteins such as fibronectin and type 1 collagen in a CHIT1 dependent manner. Furthermore, the fibrotic responses were exaggerated in mice lungs in which both CHIT1 and TGF-β1 were expressed simultaneously compared to mice in which each factors were expressed individually (94). CHI3L1 has been implicated in fibrosis and inflammation, particularly in diseases characterized by tissue remodeling. Research suggests that CHI3L1 plays an important role in fibroproliferative responses, with increased expression observed in alveolar macrophages and bronchiolar epithelial cells adjacent to fibrotic lesions (95).

As evident from the presented information, chitinases play a role in fibrosis. However, for a deeper understanding of their impact, further research is necessary. Continuing the investigation of chitinase functions in the context of fibrosis is crucial to uncover their potential roles and contribute to our overall comprehension of these processes.

## 3 *In vitro* test systems for analysis of interaction between implant materials and macrophages

The purpose of using cell system models for the analysis of implant materials is to evaluate the complex interactions between cells and implant in controlled laboratory conditions. Various cell types can be used for this purpose, including murine cell lines such as RAW 264.7 and J774A.1, primary bone marrow-derived murine macrophages (BMDM), human macrophage cell lines (THP-1, U937) and human primary monocyte-derived macrophages. It is crucial to emphasize that every cell system model has inherent limitations, and selection of cell models depends on the medical implant application and the type of tissue that the material will interact with.

### 3.1 Murine cells lines

The use of murine cell lines, such as RAW 264.7 and J774A.1 (**Table 2**), is an important tool for analyzing the effects of materials intended for implantation. These types of cell lines are widely employed to study immune responses and inflammation during the testing of implant with different compositions and modifications (96).

**Table 2:** Test systems for analysis of implant materials with murine macrophages cell lines.

Murine macrophage models	Model parameters	Plate size	Material tested	Method detection/Readout	Biological effect	Results	References
Murine сell lines							
**RAW264.7**	Cultured on TiO2 Stimulation with LPS for 24h and 48h for ELISA	75 cm^2^ flask 5×10^5^ cells/ml	TiO_2_ Coating acid etching (SLA)	IL-1β, TNF-α, IL-6, MCP1, MIP1α (all by ELISA)	Surface SLA of Ti stimulated of IL-1β, TNF-α, IL-6, MCP1, MIP1α	SLA surface of Ti, modulated expression of pro inflammatory cytokine and chemokine by macrophages	(96)
	Cultured on TiO_2_ 4 and 24 h for SEM 4, 24, 48 and 72 h for cell viability 24 and 72 h for RT-PCR and ELISA	24-well plates 1.5 × 10^5^ cells/cm^2^	TiO_2_ nanotube Layers with different diameters (30, 70 and 120 nm)	Cell Viability (MTT); Cell morphology (SEM) BMP-2 and TGF-β (RT-PCR and ELISA)	Ti nanotube layers with 30 and 70 nm exhibited more cell viability than 120 nm; Ti nanotube layers elongated cell morphology; Ti nanotube layers with 120 nm stimulated BMP-2; Ti nanotube layers did not affect TGF-β secretion	Ti nanotube layers with various diameters affected macrophage viability and bone formation	(97)
	Cultured on TiO2 48h for Immunofluorescence (IF) staining, ELISA, RT-PCR and Flow cytometry	6-well plates 1 × 10^4^ cells/ml	TiO_2_ honeycomb-like	IL-1β, TNF-α IL-4, IL-10 (all by ELISA); CD206, MBP-2 (IF, Flow cytometry)	TiO2 honeycomb-like stimulated secretion of IL-4, IL-10, CD206 and MBP-2; TiO2 honeycomb-like suppressed secretion of IL-1β, TNF-α	TiO2 honeycomb-like modulated macrophage polarization, cytokine secretion and promote bone regeneration	(98)
	Cultured on ZnONPs Cultured on ZnHNPs +PEEK Cultured on ZnHNPs +PE 6 and 24 h for RT-PCR and ELISA	96-well plates 1×10^3^ cells/ml	ZnO nanoparticles (NPs); Polyether-ether-ketone (PEEK); Highly cross-linked polyethylene (PE)	Cell Viability (CCK-8 kit) IL-1β, TNF-α, IL-6 (all by RT-PCR and ELISA) COX-2 by WB	ZnO NPs are not cytotoxic; ZnHNPs +PEEK suppressed IL-1β, TNF-α, IL-6 and COX-2; ZnHNPs +PE suppressed IL-1β, TNF-α,IL-6 and COX-2	ZnO NPs inhibited polymer wear particle-induced inflammation	(99)
	Cultured on TiPs CoPs 0, 3, 6, 12, or 24 h for ELISA 24 h for WB	6-well plates 1 × 10^6^ cells/ml	TiPs CoPs	IL-1-β and TNF-α (ELISA); SIRT-1 (WB)	TiPs and CoPs stimulated IL-1β, TNF-α; TiPs and CoPs decreased SIRT1 expression	TiPs and CoPs modulated inflammatory responses in macrophages via downregulation of SIRT1-NF-κB pathway	(100)
	Cultured on TiPs Stimulation with Bortezomib (Bzb) for 0,6,12,24,48 h.	96-well plates 3×10^5^ cells/ml	TiPs	Cell Viability (MTT); IL-1β, TNF-α, IL-6, IL-10, MCP-1, iNOS, and COX-2(all by PCR and ELISA)	Ti stimulated IL-1β, TNF-α, IL-6, MCP-1, iNOS, and COX-2. Bzb suppressed IL-1β, TNF-α, IL-6, MCP1, iNOS, COX-2, and stimulated IL-10	Bzb attenuated Ti-induced inflammation in macrophages	(101)
**J774A.1**	Cultured on Ti disks Stimulation with LPS for 24, 48, 72 h 24, 48, 72 h for RT-PCR	25 cm^2^ flask 1×10^5^ cells/ml	Ti disks grit-blasted/acid rough surface	IL-1-β, IL-6, IL-10 (all by RT-PCR); NO by colorimetric reaction with Griess reagent (Microplate spectrophotometer)	Ti grit-blasted/acid rough surfaces w/о LPS stimulated IL-1β expression and suppressed IL-6 expression; Ti grit-blasted/acid rough surfaces w/о LPS did not affect IL-10 expression; LPS stimulated expression of IL-1β, IL-6 and NO production and did not affect IL-10 expression	Ti surface topography modulated expression of proinflammatory cytokines by macrophages and involved NO pathway.	(102)
	Cultured on cpTi Stimulation with LPS for 24h 0-72 h for SEM 24,72h for RT-PCR		Polished, machined, and grit-blasted cpTi surface	Macrophage adhesion by SEM TGF-β1 and BMP-2 by RT-PCR	cpTi stimulated macrophage adhesion cpTi surface affected BMP-2 expression by time depended manner	Ti stimulated macrophage surface-specific osteoinductive signals during bone formation	(75)
	Incubated with TiAlV coating with HA Stimulation with LPS for 24h 6 and 24 h for RT-PCR and ELISA)		TiAlV coating with HA	TGF-β and BMP-2 by RT-PCR and ELISA	HA coating on TiAlV did not induce BMP-2 and TGF-β in unstimulated macrophage; LPS-activated macrophages increased level of TGF-β, but not BMP-2 in the presence HA coating on TiAlV	Bone-inductive effects of HA coating not be dependent on macrophage BMP-2 and TGF-β	(103)

These cells enable controlled laboratory experiments to assess parameters such as morphology, size, viability, cytokine levels, and gene expression. Researchers found that changes in the surface topography of the materials influences macrophage behavior and the production of inflammatory cytokines (98).

The RAW 264.7 cell line is a murine macrophage lineage derived from a tumor in a male mouse exposed to the Abelson leukemia virus. Using this cell line, Ali K. Refai et al. demonstrated that the topography of titanium surfaces significantly influences macrophage activation and their secretion of pro-inflammatory cytokines and chemokines (96). Macrophages attached to rough surfaces (acid etching and SLA) without stimulation increased the secretion of TNF-α. For macrophages stimulated with LPS, the roughest surface (SLA) led to higher levels of IL-1β, IL-6, and TNF-α at 24 and 48 hours compared to all other surfaces (96). This suggests that surface topography can modulate the expression of anti-inflammatory cytokines and chemokines by macrophages over time.

In the study by Sun et al., RAW 264.7 macrophages were cultured on layers of TiO2 nanotubes, and their morphology, adhesion, viability, and expression of BMP-2 and TGF-β1 were assessed *in vitro* (97). The study showed that macrophages grown on larger nanotube layers (120 nm) had elongated morphology and weak adhesion to the nanotube layers compared to control disks after four hours of incubation. Interestingly, macrophages remained viable on smaller nanotube layers (30 and 70 nm) even after 24 hours of incubation. Another significant finding was that increasing the nanotube diameter led to enhanced BMP-2 mRNA expression and increased BMP-2 protein secretion (97). This confirms that the TiO2 nanotube surface can influence BMP-2 expression in macrophages, potentially contributing to bone formation during regeneration.

Yizhou Zhu et al. demonstrated that the topography of TiO2 surfaces resembling honeycombs can influence macrophage polarization, a process in which macrophages transition between different phenotypes (98). Researchers created four scales of TiO2 structures resembling honeycombs on titanium substrates to study the cellular behavior of RAW 264.7 macrophages and their immunomodulation on osteogenesis. They found that reduced-scale TiO2 structures significantly activated the anti-inflammatory macrophage phenotype (M2). This was evidenced by the 90 nm diameter sample inducing the highest expression of CD206, IL-4, IL4-10, and releasing the greatest amount of BMP-2. The study suggests that by manipulating the surface topography of biomaterials, macrophage polarization can be controlled, enhancing implant osseointegration (98).

Meng et al. identified that ZnO nanoparticles can reduce inflammatory osteolysis by regulating the MEK-ERK-COX-2 signaling pathway (99). They found that ZnO nanoparticles inhibit MEK and ERK activation, leading to a reduction in COX-2 production. This decrease of COX-2 production results in reduced inflammation and bone resorption (99).

By modulating the inflammatory pathway, it is possible to reduce inflammation and alleviate symptoms of implant failure. Deng Z. et al. found that TiPs and CoPs can induce an inflammatory response during aseptic loosening through SIRT1-deacetylated NF-κB, causing its activation and subsequent inflammatory response (100). Mao et al. investigated the influence of bortezomib on inflammation modulation (101). RAW 264.7 cells grown with titanium particles and bortezomib showed increased expression of several inflammatory cytokines and enzymes, such as TNF-α, IL-1β, IL-6, MCP1, iNOS, and COX-2. In contrast, bortezomib treatment significantly reduced the expression of these inflammatory molecules in RAW 264.7 cells and induced IL-10 expression (101). These data suggest that bortezomib may inhibit inflammation induced by titanium particles in these cells.

J774A.1 cells is a cell line derived from the ascites of an adult female mouse with reticulum cell sarcoma. This cell line is not extensively utilized for investigating the immunological response to materials. The impact of titanium surface topography on the polarization, production of inflammatory cytokines, and nitric oxide in J774A.1 macrophages was elucidated by Tan et al. (102). Their study revealed that the topography of titanium surfaces can directly influence macrophage polarization, subsequently affecting the production of inflammatory cytokines and nitric oxide. Takebe et al. demonstrated that titanium surface topography has the potential to alter the morphology of J774A.1 macrophages, and these changes in cell shape could potentially impact the behavior and function of the cells (75). Additionally, titanium surface topography was found to modulate the expression of BMP-2 in J774A.1 macrophages, a protein crucial in bone remodeling. In contrast, Jakobsen et al. investigated the effects of hydroxyapatite (HA) coatings on the secretion of TGF-β and BMP-2 in murine J774A.1 macrophages (103). J774A.1 cells were exposed to TiAlV coating with or without HA, and the secretion of TGF-β and BMP-2 was monitored over time. The HA coatings did not significantly enhanced the secretion of TGF-β and BMP-2 in macrophages. However, these coatings did induce a pro-inflammatory cytokine response (103).

In conclusion, murine macrophage cell lines remain widely used for biomaterial testing. However, there are inherent limitations associated with these cells. For instance, RAW 264.7 cells exhibit genetic instability, potentially leading to alterations in cellular phenotype and function, thereby introducing variability that may impact experimental outcomes (104). Furthermore, the temporal utility of cell lines is constrained by their finite capacity for cell division. Additionally, the behavior of murine macrophages in biomaterial and inflammatory models may not fully align, posing challenges in the interpretation of experimental results. The murine origin of these cells imposes limitations in terms of data representation and interpretation within the broader context of biological systems.

### 3.2 Murine primary cells

BMDM provide a powerful tool for analyzing implant materials and enable the assessment of both pro-inflammatory and anti-inflammatory responses. The use of BMDM allows for the modulation of inflammation and implant-associated infections (**Table 3**). Using this cell model, Pearl et al. hypothesized that implant wear debris particles may act as pathogen-associated molecular patterns (PAMPs) or damage-associated molecular patterns (DAMPs), activating macrophages through TLR signaling (105). This activation leads to the secretion of TNF-α. For example, inhibiting the MyD88 protein, which plays a role in the TLR signaling pathway, reduces TNF-α production in response to polymethylmethacrylate (PMMA) particle-induced inflammation in BMDM (105).

**Table 3:** Test systems for analysis of implant materials with Primary bone marrow-derived murine macrophages.

Murine macrophage models	Model parameters	Plate size	Material tested	Method detection/Readout	Biological effect	Results	References
Primary bone marrow-derived murine macrophages (BMDM)							
C57BL/6 wild type (WT), MyD88^-/-^ and TRIF^-/-^ mice	Stimulation with M-CSF for 7 day 24 h for RT-PCR and ELISA Particle-induced osteolysis	24 well-plates 8×10^5^ cells/ml	PMMA particles (Polysciences)	TNF-α by RT-PCR and ELISA; Particle-induced osteolysis by micro-computed tomography	PMMA particles suppressed TNF-α in MyD88^-/-^ and stimulated in TRIF^-/-^ macrophages; MyD88-/- mice developed less PMMA particle-induced osteolysis than WT mice	Response to PMMA particles was dependent in part on MyD88, as part of the TLR signaling pathway	(105)
C57BL/6 mice 6 and 25 months	M-CSF for 5 day IFN-γ Simvastatin (SIMV) for 1 and 6 day for 1 and 6 day for RT-PCR	0.5 ×10^6^ cells/ml	Biomimetic calcium phosphate coating (bCaP)	IL-1β, Nos 2, Cxcl 11, Ccl 17,Arg 1(all by RT-PCR)	bCap stimulated IL-1β, Nos2, Cxcl1; bCaP with SIMV suppressed IL-1β, Nos2 and Cxcl11expression; bCaP with SIMV elevated Ccl 17,Arg1expression	bCaP stimulated proinflamatory responses. SIMV modulated inflammatory response in BMDM	(106)
6–12 week-C57BL/6 mice	Stimulation with M-CSF for 6 day IFN-γ/LPS for 72 h 24,48, 72 h for ELISA S. epidermidis (SE RP62A cells) for phagocytosis	24 well-plates 2×10^5^ cells/ml	Glass coverslips with ligand presented surface Biotin-PEG	IL-12 by ELISA Phagocytosis by microplate reader	Unmodified PEG surface did not stimulate IL-12 IFN-γ/LPS inhibited Il-12 in BMDM cultured on Biotin-PEG; Biotin-PEG stimulated bacterial killing	Biomaterial surfaces with ligands stimulated M1 macrophage and might be involved in implant-associated infections.	(107)

Alhamdi et al. explored a novel approach to control macrophage activation, especially in the context of bone healing in older adults (106). The researchers developed a biomimetic calcium phosphate coating (bCaP) that physically and temporally separated a pro-inflammatory stimulus such as IFNγ and a reparative stimulus like simvastatin (SIMV). The bCaP coating stimulated the expression of anti-inflammatory genes (IL-1β, Nos 2, Cxcl 11) in BMDM and reduced the expression of Ccl17 and Arg1. Conversely, the bCaP coating in the presence of SIMV stimulated the expression of Ccl17 and Arg1, as anti-inflammatory markers of macrophages, and reduced the expression of IL-1β, Nos 2, Cxcl 11 in BMDM. The study provided promising evidence that SIMV could be used to control macrophage activation, potentially improving bone healing (106). However, further research is needed to fully understand the involved mechanisms and explore the potential clinical applications of this approach.

Park et al. modulated a murine *in vitro* system to investigate the role of M1 macrophages in interactions with Staphylococcus epidermidis-associated implant infections (107). In this study, a biotin-PEG-based substrate was used. The unmodified PEG surface did not stimulate IL-12 production in BMDM. However, biotin-PEG promoted a decrease in IL-12 secretion in BMDM and stimulated bacterial killing (107).

The C57BL/6 mouse strain is one of the most widely used for material testing utilizing BMDM. However, some published data suggested that C57BL/6 mice exhibited high level of innate and adaptive immune responses (108, 109). It may exhibit different behavioral and physiological responses and lead to inaccurate results and misleading conclusion.

### 3.3 Human cell lines

Among human cell lines, the monocyte cell line derived from peripheral blood (THP-1) is frequently used as a macrophage model in studies investigating the healing processes of implants (**Table 4**). This ensures biological relevance. Additionally, the use of the THP-1 cell line provides a relatively straightforward and standardized method, ensuring reproducibility. Cell lines can be induced into macrophages using Phorbol-12-myristate-13-acetate (PMA), and both stimulated and non-stimulated cell lines are utilized. Another commonly employed cell line is the U937 monocyte cell line derived from human bone marrow (**Table 4**), which can also be induced into macrophages. Both cell lines serve as models for studying immune processes and inflammation in the context of tissue healing.

**Table 4:** Test systems for analysis of implant materials with human macrophage’s cell lines.

Macrophage models	Model parameters	Plate size	Material tested	Method detection/Readout	Results	Biological effect	References
THP-1	Phorbol-12-myristate-13-acetate (PMA) stimulated and PMA-free	12.5-cm^2^ sample flasks 5x10^5/^ml	Alumina ceramic particles ratios of 1:500 and 1:2500 particle size 0.8 µм and 1.3 µм. Pure titanium particles with cell-particle ratios of 1:100 and 1:500 particle size of 2 µм	TNF-α; RANK Osteoprotegerin(OPG) (RT-PCR) Viability of the macrophage-like cells(MTT assay)	Alumina ceramic particles increased expression of TNF-α, RANK and OPG. Pure titanium particles decreased TNF-α, RANK and OPG.	Positive correlation between particle concentration and cell mortality for the titanium and ceramic particles. Concentration of the titanium particles was a significant factor influencing the expression of RANK, TNF-a, and OPG	(110)
	Didn’t stimulated	12-well 5х10^5/^ml	Aluminium oxide with particle size 0.5–50 μm^3^ zirconium oxide with particle size 0.5–50 μm^3^	IL-1β (ELISA) IL-8, CCL2, CCL3, CCL4 (RT-qPCR and ELISA) Cell viability(XTT)	All types of particles increased expression of IL-1β, IL-8, CCL2, CCL3, CCL4.	Aluminium and zirconium oxide cause proinflammatory phenotype in THP-1. Oxides no significant change in cell viability	(1)
	PMA-stimulated and without PMA stimulation	96-well	Titanium particles(Ti) 60–80 nm or 100 nm and zirconia particles(Zr) of 2 μm	IL-1β, IL-6, (ELISA) Cell viability (aluminescence assay in luminometer)	Ti particles decreased viability of THP-1 cells, Zr particles decreased lower cells viability. Level of IL-1β and Il-6 was equal for all groups	Ti and Zr particles have detrimental effects on cell viability	(111)
	PMA-stimulated and primed with Lipopolysaccharides (LPS) from Escherichia coli	96-well 5x10^6/^ml	soluble cobalt, cromium, titanium and molibden	IL-1α, IL-1 β, IL-2, IL4, IL-6, IL-8, IL-10, IL-12, IL-17a, IFNγ, TNF-α (ELISA) and granulocyte–macrophage colony-stimulating factor (GM-CSF)- ELISA	Ti ions increased e[pression of IL-1 β, IL-6, IL-8, IL-10, IFNγ, GM-CSF. All samples didn’t change expression of IL-1α, IL-2, IL4, IL-12, IL-17α, TNF-α.	Ti ions stimulated inflammasome activation in human macrophages.	(112)
	PMA stimulated	1 x 10^5^/cm2 seeded on the experimental discs	Titanium discs coated with hydroxyapatite (Ti-HA) and β-tricalcium phosphate (Ti-β-TCP)	TNF-α, TGF-β (ELISA) M1: CXCL11, indoleamine 2,3-dioxygenase (qPCR); M2: MCR-1, CCL13 (qPCR)	Ti-HA, Ti-β-TCP significantly upregulated TNF-α, TGF-β cytokine secretion and marker gene expression of macrophages on HA and β-TCP coatings.	Ti-HA induced an earlier M1 macrophage polarization but more M2 phenotype potency than Ti-β-TCP.	(113)
	PMA - stimulated	12-well 2-10x10^5^/ml	Magnesium particles 31.02 μm	IL-1β, TNF-α, IL-10 (qPCR, ELISA) CD86 and CD206, CCR7 (flow cytometry)	Mg particles decreased expression level of IL-1β, TNF-α, IL-10, CCR7 and increased CD206, CCR7	Mg particles could convert macrophages from M0 to M2 phenotype.	(3)
	Stimulated with 12-O-tetradecanoyl phorbol 13-acetate (TPA)	6-well 2х10^5^/ml	TiO_2_ particle size 0.45 - 0.26µm and commercially pure Ti particles 3.32-2.39 µm	IL-6, GM-CSF, OPG (ELISA)	GM-CSF was not detected in all samples. OPG, Il-6 expression increased in samples with TiO_2_ and Ti particles	TiO2 particles increased the levels of IL-6 when applied at the dose of 50 ng/cell while Ti samples was enough to stimulate the release of this cytokine at 5 ng/cell.	(114)
U937	PMA and Vitamin D_3_ stimulated	6 well I x 10^6^/ml	Alumina powder particle size of 0.5 µm or 1.5 µm	IL-1α, IL-1β, IL-8, IL-10 and TNF-α (RT*PCR) Cell viability (confocal microscopy imaging)	Alumina particle increased expression of IL-1α, IL-1β, IL-8, IL-10, TNF-α.	Both sized particles weren’t causing cellular death, but increased inflammatory effects	(115)
U937	PMA-stimulated	24-well, 1x10^5/^ml	Cellulose nanofibril (CNF) porous scaffolds	IL-1β, IL-2, IL-6, IL-8, Il-12, IFN-γ, TNF-α, MCP-1, MIP-1α, MIP-1β CXCL-1 and M-CSF, GM-CSF, FGF, VEGF IL-1Ra, IL-4, IL-10, IL-13(ELISA) Cell viability (live/dead stanning)	CNF Scaffolds increased expression of IL-2, IL-6, IL-8, Il-12, IFN-γ, TNF-α, MCP-1, MIP-1α, MIP-1β, CXCL-1, M-CSF, GM-CSF, FGF, VEGF and didn’t change IL-1Ra, IL-4, IL-10, IL-13	CNF scaffolds supported production of anti-inflammatory cytokines IL-1β increase	(116)
U937	Stimulated with phorbol-12,13-dibutyrate (PDBu) and Vitamin D_3_	24-well 1x 10^5^/ml	HA and TCP samples diameter 15 mm	Cell viability(WST-1 assay) Osteoclast marker – TRAP (Histochemical staining)	Cell viability on all samples was equal. TRAP-positive multinucleated cells formed on HA and TCP surfaces	There was no significant difference between the samples	(117)

THP-1 and U937 cells are often used in similar experiments but may exhibit some differences in responses to various stimulants, attributed to their distinct origins and histories.

Employing various methods (ELISA, RT-PCR, XTT, flow cytometry, etc.), these cell models allow for the examination of cell survival in immune peri-implant tissues, their adhesion and migration capabilities (**Figure 1**). Most commonly, the models focus on the production of inflammatory cytokines. In the following study soluble cobalt, chromium, titanium, and molybdenum were administered to PMA-stimulated THP-1 macrophages, which were previously primed with LPS (110–112, 114). Studies have demonstrated a significant increase in pro-inflammatory cytokines and GM-CSF induced by titanium (Ti) ions, indicating an inflammatory response. Interestingly, the levels of other cytokines, specifically IL-1α, IL-2, IL-4, IL-12, IL-17α, and TNF-α, remained largely unaltered. Furthermore, titanium ions were discovered to stimulate inflammasome activation in human macrophages, revealing findings regarding their immunomodulatory potential (110–112, 114).

In study PMA-stimulated THP-1 macrophages were grown on titanium discs that had been coated with hydroxyapatite (Ti-HA) and β-tricalcium phosphate (Ti-β-TCP) (113). Both coatings led to a significant increase in cytokine secretion of TNF-α and TGF-β, as well as expression of marker genes for M1 and M2 macrophages. It is worth noting that Ti-HA coating caused an earlier polarization of M1 macrophages and showed greater M2 phenotype potential compared to Ti-β-TCP. In another study THP-1 macrophages stimulated with PMA were exposed to magnesium particles. The findings indicated that magnesium particles decreased the production of IL-1β, TNF-α, IL-10, and CCR7, while improving the expression of CD206 and CCR7. This suggests that magnesium particles have the capability to transform macrophages to an M2 type (3).

In U937 cells, which were cultured on porous cellulose nanofibril (CNF) substrates, the expression of several pro-inflammatory cytokines was significantly increased, while there was no significant change in anti-inflammatory cytokines (116). On the fourth day of *in vitro* culture, CNF scaffolds demonstrated significantly increased expression of anti-inflammatory IL-1Ra and IL-10 genes. It is noteworthy that the production of inflammatory cytokines IL-1β, IL-6, MCP-1, MIP-1α, CXCL-1, and M-CSF was significantly lower in CNF scaffolds, indicating an early and weak inflammatory response (116). U937 cells, exposed to PDBu and Vitamin D3, were cultivated on samples of hydroxyapatite (HA) and tricalcium phosphate (TCP) (117). Cell viability was consistent across all samples, and multinucleated cells that tested positive for TRAP were present on both HA and TCP surfaces, with no significant distinction.

However, it is essential to note that such models may not fully replicate the complex interactions within the organism, and results may be context-dependent. Therefore, they should be complemented and validated with data obtained from more sophisticated research systems. Such as three-dimensional (3D) cell models are an alternative to two-dimensional (2D) cell culture models that have the potential to be more physiologically relevant. Commonly used models include the generation of spheroids and organoids, bio scaffolds based on synthetic (polyacrylamide or polyethylene glycol (PEG) or natural polymers (gelatin, collagen) (118–120). 3D bioprinting techniques are outstanding for scaffold fabrication due to their ability to create porous structures with interconnected cells and growth factors for *in vitro* and *in vivo* evaluation as preclinical assessments (121). Organs-on-a-chip (OOCs) technology has been increasingly used to study the immune system, providing a more realistic *in vitro* environment compared to traditional 2D cultures. OOCs technology is used in the study of bone marrow, spleen, can also modulate inflammation (122–124).

These studies collectively emphasize the diverse nature of macrophages responses to a broad range of stimuli. Understanding this plasticity is crucial in uncovering the complexities of macrophage function in different physiological and pathological settings, highlighting the significant role of cellular models in immunomodulatory research.

### 3.4 Human primary macrophages cells lines

To understand the regulation of immune and inflammatory responses with implants, an important model for researchers is monocyte-derived macrophages differentiated on biomaterial surfaces.

In contrast to the aforementioned cell lines, human primary macrophages more accurately reflect the physiological characteristics of human tissues, as they are directly derived from the human organism. This ensures a more reliable reproduction of real conditions in tissues. Additionally, primary macrophages maintain the heterogeneity inherent in the human body. Therefore, investigating implant materials on patient-derived cell lines may enable the prediction of the inflammatory response to the implant within the patient’s body (125). However, acquiring human primary macrophages is a labor-intensive process, and differences between donors persist. When utilizing human primary macrophages, it is also possible to trace immune reactions to implant installation (**Table 5**). The investigation into implant using monocyte-derived macrophages M0, M1, and M2, differentiated on PAR/HA and PAR/HA+CAT surfaces, illustrated that these substances decrease TNF-α production and CD206 level. This suggests the implant have the potential to restrict inflammatory processes (126). However, the use of these materials also resulted in an increase in the level of IL6 in some donors, revealing the complexities in regulating immune responses (126).

**Table 5:** Test systems for analysis of interaction of implant materials with human primary macrophages.

Macrophage models	Model parameters	Plate size	Material tested	Parameter/Method	Results	Biological effect	Reference
Monocyte-derived macrophages M0, M1 and M2 differentiated on biomaterial surfaces	M0, M1 (IFNγ-stimulated); M2 (IL4-stimulated) SFM supplemented by Dexametasone 10^-8^M	24-well plates 1×10^6^/ml	PAR/HA films (polyarginine (PAR) and hyaluronic acid (HA); PAR/HA+CAT films (functionalized by embedding of catestatin)	TNF-α (ELISA); CCL18 (ELISA); IL6 (ELISA) CD206 (confocal microscopy)	PAR/HA and PAR/HA+CAT suppress production of TNF-α PAR/HA and PAR/HA+CAT slightly increased IL6 in part of donors PAR/HA and PAR/HA+CAT decreased CD206 expression	PAR/HA and PAR/HA+CAT Decreases proinflammatory potential of both M1 and M2	(126)
Monocyte-derived macrophages M0, M1 and M2 differentiated on biomaterial surfaces	M0, M1 (IFNγ-stimulated); M2 (IL4-stimulated) SFM supplemented by Dexametasone 10^-8^M	24-well plates 1×10^6^/ml	PLA films (polylactic acid) BGD1,2 and 3 (PLA films with surface modifications	TNF-α (ELISA); CCL18 (ELISA); CD206 (confocal microscopy) Stabilin-1 (confocal microscopy)	BGD1, BGD2, and BGD slightly increased TNF-α PLA decreased CCL18 in some donors BGD1, BGD2, and BGD3 increased CCC18 in some donors BGD1, BGD2, and BGD3 effects on CD206 and stabilin-1 were donor-specific	Model system enables prediction of patient-specific reactions	(30)
Monocyte-derived macrophages M0, M1 and M2 differentiated on biomaterial surfaces	M0, M1 (IFNγ-stimulated); M2 (IL4-stimulated)	12-well plates 2×10^6^/ml	PLA-based scaffolds with hyaluronic acid	Cell Viability Assay (Alamar Blue) TNF-α, IL-6, IL-8, IL-1β, IL-10, IL-1ra, CCL18, TGFβ, MMP7, and MMP9 (ELISA)	Level of Il-1ra, CCl18 increased in M1; Increased level of MMP9, IL-8 Level of TGFβ, TNF-α and MMP7 decreased	In certain samples, IL-6, IL-8 secretion increased on day 6, TNFα elevated фаеук6 hours of co-culture. One sample showed reduced MMP7 expression in M0 macrophages. Despite increased IL1ra, another sample’s M0 and M1 macrophages released higher levels of IL6 and IL8 when co-cultured with PLA-HA. Some samples did not display specific reactions to the materials	(125)
Monocyte-derived macrophages M0 differentiated on biomaterial surfaces	X-VIVO supplemented with 1 ng/mL M-CSF and 10^−8^M dexamethasone	24-well plates 1×10^6^/ml	PCL (poly(ϵ-caprolactone) scaffolds modified by Reactive Magnetron Sputtering	Cell Viability Assay (Alamar Blue) Endocytosis of acLDL-Alexa48 ROS production	No effect on cell viability PCL scaffolds had no inhibitory effect on ROS Modified scaffolds decreased ROS	Scaffolds are nontoxic, retain scavenging function, and suppress acute inflammatory response	(127)
Monocyte-derived macrophages M0 differentiated on biomaterial surfaces	RPMI supplemented with 50 ng/mL M-CSF. 200 ng/mL LPS	24-well plates 2.5×10^5^ macrophages per patterned hydrogel (≈1.32×10^5^ macrophages/cm^2)^	GelMA (gelatin methacryloyl) micropatterning	Morphology by F-actin staining PhalloidinAlex488 quantified by IMSTAR automated fluorescent microscopy TNF-α, IL-12, IL-1β, CCL18, IL-1RA (all by ELISA). Phagocytosis of Alexa Fluor 488-labeled zymosan by flow cytometry. Microarray Transcriptome Analysis	GelMA hydrogels decreased size of macrophages and cytoplasm to nucleus ratio with no effect of micropatterning. No significant effects of phagocytosis except micropillars. Microgroove/ ridge and micropillar patterning on GelMA significantly reduced production of TNF-α. Micropillars had the greatest impact on macrophage gene expression	Unbiased screening of macrophage responses to biomaterials revealed new processes affected by micropatterning	(128)
Monocyte-derived macrophages M0, M1 and M2 differentiated in the presence of TiNPs	M0, M1 (IFNγ-stimulated); M2 (IL4-stimulated); SFM with 5mM glucose supplemented by Dexametasone 10^-8^M	12-well plates 1×10^6^/ml	TiNPs	Transcriptome (Affymetrix chips) GDF-15, stabilin-1 (RT-PCR) GDF-15 (ELISA) Endocytosis of cLDL-Alexa488 (flow cytometry, confocal microscopy)	TiNPs altered expression of 5098 genes in M1 and 4380 genes in M2 TiNPs upregulated GDF-15 and suppressed stabilin-1 TiNPs suppressed stabilin-1 mediate endocytosis	TiNPs Elevate GDF15 levels by stimulation of its production and suppression of its clearance.	(62)
Monocyte-derived macrophages M0, M1 and M2	M0, M1 (IFNγ-stimulated); M2 (IL4-stimulated); SFM supplemented with 10 ng/ml M-CSF and 1% PSA 1, 4, and 6 days	24-well plates 1×10^6^/ml	porcine cartilage DECM DNSCn as discs (*diam*. = 5 mm, *height* = 1 mm) or particles (DNSCp). =	Viability/Cytotoxicity Assay IL-1β, TNF-α, CCL18, (RT-PCR) CD38, CD206 (flow cytometry), CCL18, IL-6, TNF-α, and IL-1β (Multiplex assay).	DNSC disks and particles did not affect viability but induced IL-1β, TNF-α and IL-6, as well as CCL18 and CD206.	Functionalization of DNSC with IL-4 was necessary to overcome mixed activation profile of macrophages	(129)

In the research conducted on polycaprolactone (PLA) implant, which analyzed several modifications of PLA films (BGD1, BGD2, and BGD3), disparate effects on M0 macrophages were observed (30). These adjustments raised TNF-α levels while also having donor-specific impacts on CCL18 (30).

For the investigation of PCL implant, M0 macrophages were subjected to micro-patterned PCL hydrogels (127). The findings suggest PCL scaffolds do not present toxicity and are capable of reducing ROS levels. This can be highly significant in curbing inflammatory responses (127).

The effects of micro-patterning on GelMA hydrogels were investigated in a study. The study found that micro-patterning had an impact on both macrophage size and TNF-α level (128). Furthermore, the researchers identified novel processes that could potentially be influenced by micro-patterning, highlighting the significance of this methodology in medical tissue engineering and implantology (128).

These studies indicate the potential of biomaterials and their modifications for regulating immune and inflammatory responses. This may provide a foundation for developing more effective and personalized strategies in medical tissue engineering and implantology.

## 4 Current 3D model for biomaterial testing

As of today, it is acknowledged that cell cultures in 2D models may not always faithfully represent the physiological complexity of tissues, which are structurally intricate, cellularly heterogeneous, and dynamically changing over time within the human body (130). Contemporary 3D cell culture models are gaining popularity due to their ability to achieve a higher degree of cell differentiation and tissue organization compared to 2D culture systems.

Various techniques exist for creating 3D cell cultures, such as spheroid culture, biopolymer scaffolds, microfluidics, and organs-on-chip, aiming to replicate and mimic *in vivo* systems (**Table 6**). Several studies have investigated the influence of 3D scaffolds made from different materials on the polarization of macrophages and immune responses to implants (**Table 6**). Almeida et al. focused on the effects of 3D-printed Polylactic Acid (PLA) and chitosan-based scaffolds on human monocyte/macrophage responses (131). PLA-based and chitosan scaffolds increased TNF-α secretion. Despite PLA-based scaffolds inducing higher production of interleukins IL-6, IL-12/23, and IL-10, chitosan scaffolds with larger porous structures and wider angles influenced cellular responses and pro-inflammatory cytokine secretion.

**Table 6:** Type of 3D model for material testing.

Year	Type of model	Matrix, scaffold	Cell types, Tissue composition	Tested materials	Analytical methods	Outcome	References
2014	3D model of inflammation	Biodegradable 3-D scaffolds	Human primary monocytes,	PLA and PEG Calcium phosphate glass(G5) Chitosan (Ch)	ELISA	PLA-based scaffolds induced IL-6, IL-10, IL-12/23; PLA/G5 based scaffolds induced IL-6; Ch based scaffolds induced TNF-α	(131)
2019	FBROC device	Gel MA	Human Primary Monocytes, THP-1, HUVECs	Ti microbeads	ELISA, IF, Cell tracker (CM Dil dye)	Ti microbeads stimulated M1 phenotype macrophage; FBROC potentially to use for investigation of personalized FBR	(133)
2020	3D Oral Mucosa Models	Four types of rods with 4 mm diameter and 8 mm length	KF6/TERT-2 human oral keratinocyte, Human oral fibroblasts, THP-1	TiZr-SLA TiZr-M ZrO_2_-M PEEK-M	PrestoBlue, Electron Microscopy, Histology	TiZr-SLA increased cell viability TiZr-M, ZrO2-M, PEEK-M induced flat cell morphology TiZr-SLA induced 3D morphology	(132)
2021	3D tracheal patch	Silicone	Human monocyte-derived macrophages	Gelatine hydrogel coated implants with cytokine cocktail M2Ct2 (IL-10, PGE-2)	ELISA RT-qPCR IF Histology	Immunomodulatory hydrogel inhibited proinflomatory response and promoted better integration implants with tissue	(134)
2022	3D model FBR and bone regeneration	Bioactive scaffolds with different pore sizes (P200,P400,P600)	Rat bone mesenchymal stem cells RAW 264.7	Polycaprolactone/polyethylene glycol/hydroxyapatite	CCK-8 Live/Dead Staining, qRT−PCR, IF, Micro-CT	P600 diminished FBR; PCL/PEG/HA bioactive ceramic scaffolds with a pore size of 600 are promising for bone regeneration.	(135)
2022	3D bone regeneration	Polyetheretherketone (PEEK)	MC3T3-E1 HUVEC	Magnesium ions (Mg^2+^)	CCK-8 IF RT-qPCR Western blotting Micro-CT Histology	Mg^2+^coated scaffold induced cell adhesion, proliferation, angiogenesis and contributed for osteointegration	(136)

Barker et al. developed a 3D oral mucosal model by combining human oral fibroblasts, OKF6/TERT-2 keratinocytes, and THP-1 cells (132). Implants (TiZr-SLA, TiZr-M, ZrO2-M, PEEK-M) were inserted into the tissue-engineered oral mucosa following a 4 mm punch biopsy. Inflammation was simulated by adding LPS from E. coli and TNF-α to the culture medium. Histological data showed that the inflamed oral mucosa model closely mimicked the *in vivo* situation, with a 3D dimensional structure comprising the connective tissue collagen layer containing fibroblasts and monocytes and a distinct epithelial layer with multi-layered stratified oral keratinocytes (132).

Barthes et al. presented a study addressing potential adverse effects of 3D-printed silicone implants, such as tracheal defect repair due to immune reactions (134). The study focused on controlling the implant/host interface using immunomodulatory coatings. The researchers designed a new cytokine cocktail composed of interleukin-10 and prostaglandin-E2, aiming to decrease adverse immune reactions and promote tissue integration by fixing macrophages into an M2 pro-healing phenotype for an extended period. The study concluded that the ability of this new immunomodulatory hydrogel to control inflammation levels once applied to a 3D-printed silicone implant has been demonstrated (134).

Li et al. described a 3D model for foreign body response and bone regeneration (30). 3D-printed scaffolds were prepared using a combination of polycaprolactone (PCL), polyethylene glycol (PEG), and hydroxyapatite, focusing on various pore sizes of the scaffolds (P200, P400, P600). This 3D model demonstrated that P600 pore size significantly reduced the foreign body response and induced a more M2 macrophage phenotype, vascular ingrowth, and new bone formation (137).

Wei X. et al. also established a 3D model for bone regeneration using Polyetheretherketone (PEEK) (138). PEEK scaffolds were manufactured via fused deposition modeling, and a polydopamine (PDA) coating chelated with magnesium ions was applied to the surface. *In vitro* results showed that the activated surface promoted cell proliferation and adhesion, contributing to osteoblast differentiation and mineralization. The released magnesium ions also promoted angiogenesis (138).

These findings suggest that the design of 3D-printed scaffolds, including material selection and control of pore size and coating, can be optimized to enhance the effectiveness of tissue engineering and regenerative medicine. Recently, microphysiological systems, known as OOCs technology, enable the study of physiological processes in the human body using modern models for diseases of the lung, liver, kidney, gut, brain, bone marrow, kidneys, tumor-on-chip, and drug testing (139, 140). Sharifi F. et al. created an *in vitro* microfluidic platform to reproduce the dynamic effects of human primary monocytes and THP-1 cell line on Ti microbeads occurring in foreign body responses (133). They proposed that this platform would be a valuable tool for studying the immune foreign bodies response.

Organoid technology is a rapidly advancing field, providing a platform for the study of cancer behavior, drug discovery and testing, disease modeling, and host-microbiome interaction (131). Bone organoids represent a novel concept in tissue engineering. Iordachescu A. et al. constructed trabecular bone organoids using primary female osteoblastic and osteoclastic cells, seeded onto femoral head micro-trabeculae (141). These cells recapitulate relevant phenotypes and functions. Once the organoids are constructed, they are inserted into a simulated microgravity bioreactor to model a pathological state of reduced mechanical stimulation. In these constructs, osteoclastic bone resorption sites can be detected, which differ in morphology in the simulated microgravity group compared to static controls. In conclusion, bone organoids represent a promising approach for studying bone regeneration and repair.

3D bioprinting is a rapidly evolving field with the potential to revolutionize biomaterial testing and tissue engineering. It involves the layer-by-layer deposition of biological materials to create complex structures that mimic natural tissues or organs (142). This technology can be employed for material testing in various ways. In the context of bone modeling, 3D bioprinting can be used to fabricate scaffolds for bone regeneration. These scaffolds can be composed of hydrogel materials, which are ideal for this purpose, due to their controllable biological and biophysical properties (143). Hydrogels can support the attachment, proliferation, migration, and differentiation of cells, crucial for the regeneration of bone tissue.

**Figure 2:**
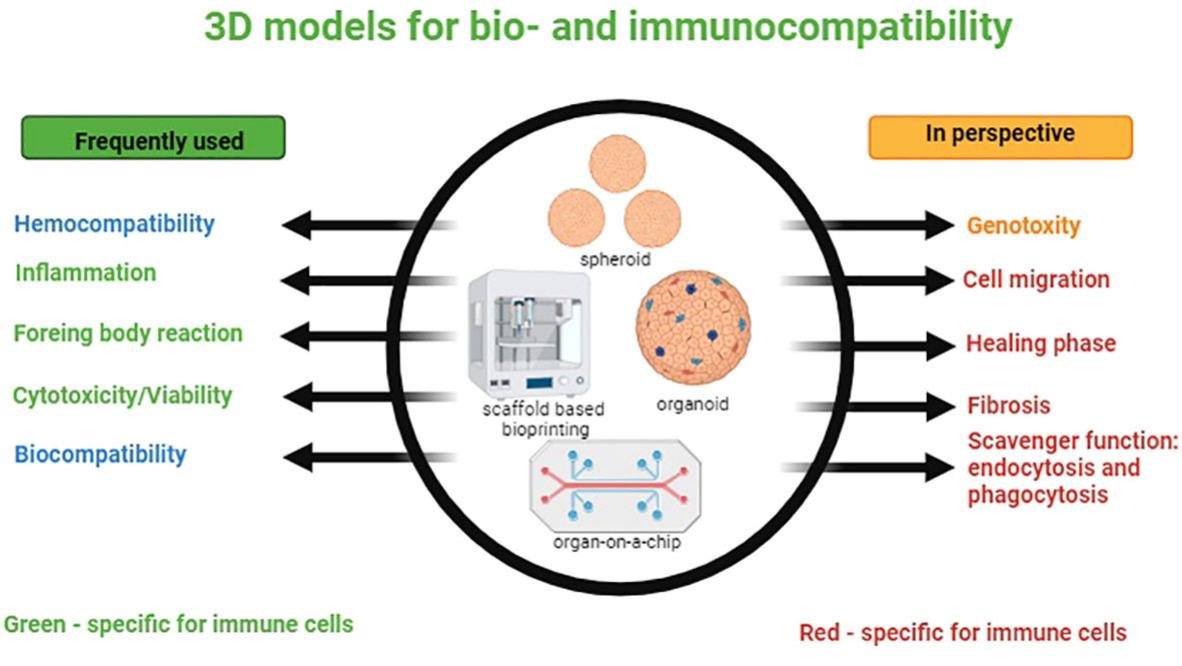
3D models for bio- and immunocompatibility. On the left-side processes frequently simulated *in vitro* models are listed; immune-specific processes are marked in green. On the right-side processes that have to be modeled in future are listed. Immune-specific processes are marked in red.

Briefly, the exploration of 3D cell culture models, with techniques like spheroid culture, biopolymer scaffolds, microfluidics, and organs-on-chip, has shown their potential for more physiologically relevant platforms compared to traditional 2D models. Optimizing 3D-printed scaffolds through material selection, pore size control, and coating is critical for improving tissue engineering efficacy. Advanced microphysiological systems, particularly OOCs offer innovative approaches for studying various organs and tissues. Bone organoids and 3D bioprinting applications in bone modeling demonstrate these technologies’ versatility in advancing understanding of bone regeneration (**Figure 2**).

In summary, the state of the art in *in vitro* and ex vivo modelling of macrophage interaction with biomaterials is primarily focused on the mechanic interactions and acute inflammatory reactions, and such modeling allows to detects first line detrimental reactions of macrophage to biomaterials. However, successful implant integration requires the escape from the chronic inflammatory scenario and also suppression al the tissue –destructive activities of macrophages that constitute a part of foreign body response. Modelling of such reaction avoiding animal experimentation is challenge for the scientific community. However, a lot of information can be obtained already in 2D primary macrophage based models, where new biomarkers predicting efficiency of healing and long-term integration of the implant can be discovered. Upgrading the models to 3D conditions will allow further approximation to the *in vivo* events.

## Author contributions

SP: Writing – original draft, Writing – review & editing. GR: Visualization, Writing – original draft, Writing – review & editing. AB: Project administration, Writing – review & editing. SC: Writing – review & editing. IA: Project administration, Writing – review & editing. VP: Project administration, Writing – review & editing. JK: Conceptualization, Supervision, Writing – original draft, Writing – review & editing, Funding acquisition.

## Glossary

**Table:**

CR3	complement receptor 3
avß3	integrin
a5ß1	integrin
αDβ2	integrin
TNF-α	Tumor necrosis factor alpha
IL-1Ra	interleukin-1receptor antagonist
IL-1β α	interleukin1 alfa
IL-1β	interleukin1 beta
IL-2	interleukin 2
IL-4	interleukin 4
IL-6	interleukin 6
IL-8	interleukin 8
IL-10	interleukin 10
IL-12	interleukin 12
IL-13	interleukin 13
IL-17α	interleukin 17alfa
IL-23	interleukin 23
MIL-4	murine Interleukin-4
IGF-1	insulin-like growth factor 1
VEGF	vascular endothelial growth factor
PDGF	platelet-derived growth factor
FGF	fibroblast growth factors
PF4	platelet factor 4
CXCL-1	Chemokine (C-X-C motif) ligand 1
CXCL2	Chemokine (C-X-C motif) ligand 2
CXCL3	Chemokine (C-X-C motif) ligand 3
CXCL4	Chemokine (C-X-C motif) ligand 4
CXCL7	Chemokine (C-X-C motif) ligand 7
CXCL8	Chemokine (C-X-C motif) ligand 8
CXCL9	Chemokine (C-X-C motif) ligand 9
CXCL10	Chemokine (C-X-C motif) ligand 10
CXCL11	Chemokine (C-X-C motif) ligand 11
CCL3 (MIP-1α)	Chemokine (C-C motif) ligand 3(macrophage inflammatory protein 1-alpha)
CCL13	chemokine (C-C motif) ligand 13
CCL18	chemokine (C-C motif) ligand 18
CCR7	c-c chemokine receptor type 7
FBGCs	foreign body giant cells
BMP-2	bone morphogenetic protein-2
RUNX2	runt-related transcription factor 2
MCP1	monocyte chemoattractant protein 1
MIP1α	monocyte chemoattractant protein 1alfa
MIP1 β	monocyte chemoattractant protein 1beta
iNOS	inducible nitric oxide synthase
IFN-γ	interferon gamma
MLPS	M lipopolysaccharide
M-CSF	macrophage colony-stimulating factor
GM-CSF	granulocyte-macrophage colony-stimulating factor
GDF-15	growth differentiation factor 15
MMPs	matrix metalloproteinases
MMP-9	matrix metalloproteinase 9
CD36	cluster of differentiation 36
CD38	cluster of differentiation 38
CD68	cluster of differentiation 68
CD163	cluster of differentiation 163
CD206	cluster of differentiation 206
MARCO	macrophage receptor with collagenous structure
Lyz2	lysozyme 2 protein
TLR	toll-like receptor
SIRT1	sirtuin 1
RANK	receptor activator of nuclear factor κB
OPG	osteoprotegerin
Alpha-SMA	alpha-smooth muscle actin
FN1	fibronectin protein
MyD88	myeloid differentiation primary response 8
TRAP	region amplified polymorphism
CHIT1	Chitinase1
CHIT3	Chitinase3
COX-2	cyclooxygenase-2
Nos2	nitric oxide synthase 2
Arg1	protein arginase1
ECM	extracellular matrix
DECM	decellularized extracellular matrix
CEP	carboxyethylpyrrole
SLA	sandblasted and acid-etched surface
Gel MA	methacrylated Gelatin
PEEK	polyether ether ketone
PMA	phorbol-12-myristate-13-acetate
PMMA	polymethylmethacrylate
PEG	polyethylene glycol
PCL	polycaprolactone
PLA	polylactic acid
PAR	polyarginine
bCaP	biomimetic calcium phosphate
HA	hydroxyapatite
HA	hyaluronic acid
TCP	tricalcium phosphate
Ch	Chitosan
SNF	cellulose nanofibril
DNSC	decellularized nasal septal cartilage
CAT	catestatin
BGD1,2,3	brilliant green dye 1,2,3
DCs	dendritic cells
NK	natural killer
BMDM	bone marrow-derived murine macrophages
OOCs	organs-on- organs-on-chip
FBROC	foreign body response-on-a-chip platform
FBR	foreign body response
ROS	reactive oxygen species
SSc	systemic sclerosis
POD	postoperative days
